# The Rate-limiting Step of DNA Synthesis by DNA Polymerase Occurs in the Fingers-closed Conformation

**DOI:** 10.1016/j.jmb.2021.167410

**Published:** 2022-01-30

**Authors:** Geraint W. Evans, Timothy Craggs, Achillefs N. Kapanidis

**Affiliations:** 1 Department of Physics and Biological Physics Research Group, Clarendon Laboratory, University of Oxford, Parks Road, Oxford OX1 3PU, United Kingdom; 2 Sheffield Institute for Nucleic Acids, Department of Chemistry, University of Sheffield, Brook Hill, Sheffield S3 7HF, United Kingdom

**Keywords:** DNA polymerase, fingers opening-closing transition, DNA synthesis, real-time single molecule kinetics, single-molecule FRET

## Abstract

DNA polymerases maintain genomic integrity by copying DNA with high fidelity, part of which relies on the polymerase fingers opening-closing transition, a series of conformational changes during the DNA synthesis reaction cycle. Fingers opening and closing has been challenging to study, mainly due to the need to synchronise molecular ensembles. We previously studied fingers opening-closing on single polymerase-DNA complexes using single-molecule FRET; however, our work was limited to pre-chemistry reaction steps. Here, we advance our analysis to extensible substrates, and observe DNA polymerase (Pol) conformational changes across the *entire* DNA polymerisation reaction in real-time, gaining direct access to an elusive post-chemistry step rate-limiting for DNA synthesis. Our results showed that Pol adopts the fingers-closed conformation during polymerisation, and that the post-chemistry rate-limiting step occurs in the fingers-closed conformation. We found that fingers-opening in the Pol-DNA binary complex in the absence of polymerisation is slow (∼5.3 s^−1^), and comparable to the rate of fingers-opening after polymerisation (3.4 s^−1^); this indicates that the fingers-opening step itself could be largely responsible for the slow post-chemistry step, with the residual rate potentially accounted for by pyrophosphase release. We also observed that DNA chain-termination of the 3′ end of the primer increases substantially the rate of fingers-opening in the Pol-DNA binary complex (5.3 → 29 s^−1^), demonstrating that the 3′-OH residue is important for the kinetics of fingers conformational changes. Our observations offer mechanistic insight and tools to offer mechanistic insight for all nucleic acid polymerases.

## Highlights

• Real-time study of DNA polymerase fingers conformations during DNA synthesis.
• DNA polymerase adopts predominantly a fingers-closed conformation during synthesis.
• The post-chemistry rate-limiting step occurs in the fingers-closed conformation.
• The 3′-OH group substantially affects the kinetics of fingers conformational changes.
• Approach well suited for multi-step reactions driven by proteins on nucleic acids.

## Graphical abstract

**Figure:**
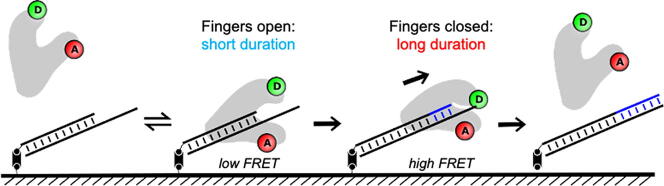

## Introduction

DNA polymerases replicate and repair cellular DNA with extraordinary accuracy, contributing substantially to the maintenance of genomic integrity in all organisms. For instance, the *Escherichia coli* DNA polymerase I mis-incorporates nucleotides only once every 10,000–100,000 nucleotides.^1^ This high fidelity is achieved by selecting nucleotides for their complementarity with a template DNA strand, whilst strongly discriminating against and rejecting non-complementary dNTPs and all rNTPs. Steps before chemical nucleotide incorporation (“pre-chemistry steps”) contribute heavily to these selection mechanisms, and have been extensively studied^2^, ^3^, ^4^, ^5^, ^6^, ^7^; of particular importance is a large conformational motion of the fingers sub-domain of the polymerase, which moves from a “fingers-open” conformation in the absence of nucleotide, to a “fingers-closed” conformation when recognising a complementary nucleotide.^8^, ^9^, ^10^, ^11^ Conversely, the polymerase enters a “partially-closed” conformation in the presence of non-complementary nucleotide.^12^, ^13^, ^14^, ^15^

Steps after chemical incorporation of a nucleotide (“post-chemistry steps”) are also important for fidelity, allowing the polymerase to check the incorporated entity, and excise mis-incorporated nucleotides, if necessary, by switching to its exonuclease mode.^16^, ^17^ The main post-chemistry steps consist of pyrophosphate product release,^18^ translocation, and fingers-opening. One or more of these steps may also be implicated in a rate-limiting “slow” post-chemistry process, which was inferred via the difference in yield between pulse-quench and chase experiments,^18^, ^19^ and also by fluorescence.^20^ Despite the significance of these post-chemistry steps for the accuracy and mechanisms of DNA synthesis, these steps have been less well studied, partially because investigating the later reaction stages of the dNTP addition cycle typically require synchronising reagent ensembles, which is very difficult to achieve. As a result, many aspects of the post-chemistry steps remain unclear; such aspects include the physical origin and purpose of the slow post-chemistry step, and the relative sequence of the translocation, fingers-opening, and pyrophosphate-release steps. Due to the rate-limiting nature of the slow post-chemistry step, this step may be instrumental in understanding the wide variance in processive polymerisation rates observed for the Klenow Fragment (KF) of the bacterial DNA polymerase I.^18^, ^21^, ^22^, ^23^, ^24^, ^25^

**Figure 1:**
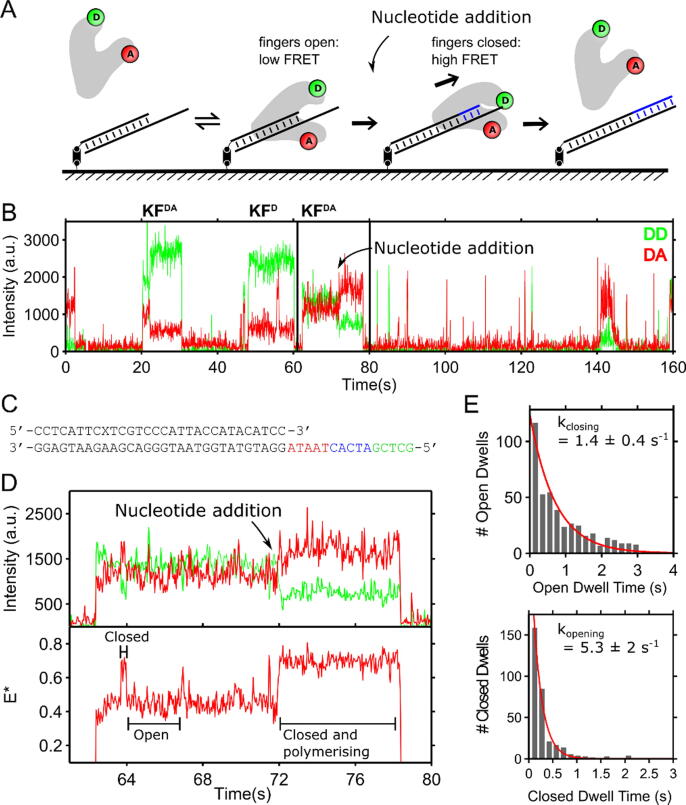
(A) Schematic illustrating the single-molecule assay used to observe the fingers conformation of KF during DNA polymerisation. Extensible DNA primer-templates are immobilised onto a glass surface (left), with donor–acceptor labelled KF in solution free to bind the DNA. Fingers conformations during nucleotide incorporation are observed via changes in FRET while nucleotides are incorporated (blue) into the primer strand, leaving an extended DNA or blunt end. (B) Fluorescence emission at a single DNA molecule over time, demonstrating KF binding to DNA via fluorescence increases. Nucleotides were added at ∼72 s, leading to a change energy transfer between donor and acceptor dyes, and afterwards lower fluorescence emission overall, indicating lower KF-DNA binding after polymerisation. Black box expanded in panel D. (C) Base-paired primer-template oligonucleotide used with the -22 position on the primer labelled with a Cy5 fluorophore (denoted by an “X”) and the 5′ terminal biotinylated for surface immobilisation. The template is designed to allow extension by 1, 5, 10 and 15 bases, depending on the nucleotide subset used. (D) Expanded black box from panel B, demonstrating the increase in FRET at 72 s caused by nucleotide addition, signifying fingers-closing of KF and polymerisation of the DNA, which occurs for 6 s, followed by dissociation of KF from the DNA. Prior to nucleotide addition the KF-DNA complex undergoes binary complex fingers-dynamics on faster time-scales. Donor-excitation, donor-emission (DD – green). Donor-excitation, acceptor-emission (DA – red). FRET efficiency (E* – red) (E) Dwells in the fingers-open and fingers-closed conformation before nucleotide addition, extracted using HMM.

In previous work, we directly monitored the conformation of the fingers subdomain of the Pol-I KF using single-molecule FRET between a donor fluorophore on the mobile portion of the KF fingers, and an acceptor on the KF thumb.^15^, ^26^ Using a Total Internal Reflection Fluorescence (TIRF) assay, we were able to monitor the fingers conformations in real-time by observing single labelled KF molecules as they transiently bound to surface-immobilised DNA template molecules (Figure 1(A)).^27^ Specifically, we monitored the fingers conformation during the pre-chemistry reaction by using inextensible (dideoxy-terminated) DNA substrates, and thereby quantified the kinetics of the polymerase fingers opening and closing, as well as the contribution of pre-chemistry steps to fidelity. However, the chain termination excluded investigation of post-chemistry reaction steps, and may have affected the pre-chemistry kinetics.

Here, we extend our smFRET observations to conformational changes of the KF’s fingers across the *entire* DNA polymerisation reaction. To achieve this, we used extensible DNA primer-templates, which permit specific numbers of nucleotide incorporations (Figure 1(A)), and performed real-time smFRET measurements. These observations allowed us to extract the rates of single-nucleotide and multiple-nucleotide polymerisations, and to show that any post-chemistry steps in the fingers-open conformation are brief, and contribute very little to the total time taken for polymerisation. Further, we show that the rate-limiting post-chemistry process occurs in the fingers-closed conformation, and may indeed be largely due to fingers-opening itself. Our method opens the door to detailed studies of post-chemistry kinetics and, more generally, helps define the physical basis of steps critical for the speed and fidelity of DNA polymerases.

## Materials and methods

### Protein and DNA preparation

The KF-FRET construct was prepared as described previously, with dye labels Cy3B on the fingers (L744C), and Atto647N on the thumb (K550C), respectively,^15^ in a Cys-free, exo-minus background (C907S, D424A). The primer-template oligonucleotides (Figure 1(C)) were synthesized and HPLC-purified by IBA Life Sciences (Germany), and then gel purified. The primer strand was 5′ biotinylated and labelled with Cy5 at the -22 position. The template oligonucleotides were synthesized by Sigma-Aldrich and gel purified.

### TIRF experiments

**Figure 2:**
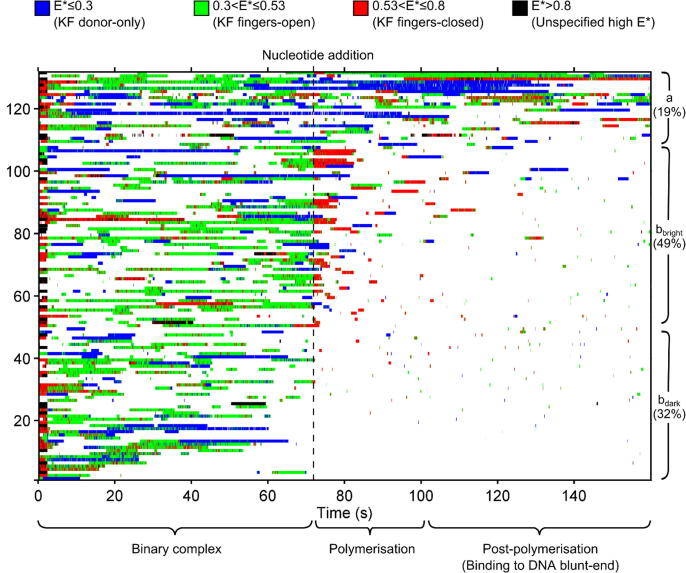
Visualisation representing the binding and conformation of KF molecules to an ensemble of ∼132 DNA molecules from a single-experiment. Each horizontal line represents fluorescence time-series at a DNA molecule (as in Figure 1(B)). Fluorescence increases caused by KF binding events are represented in colour, with the colour reflecting the FRET efficiency at each time point, as indicated in the figure. These FRET values correspond to the following states: Green: KF fingers-open. Red: KF fingers-closed. Blue: Donor-only KF. Black: Unspecified high FRET. Binding events before nucleotide addition (dotted line) are seen to be predominantly open (green), consistent with binary complex behaviour. A large increase in the number of fingers-closed KF molecules (red) is seen after nucleotide addition, which we interpret as KF molecules polymerising DNA. Decreased KF-DNA binding is seen subsequent to these polymerisations. The period between 0 and 3 seconds is due to the activated red laser used to localise the DNAs. Group (a): DNA molecules which do not show decreased KF binding after nucleotide addition. Group (b): DNA molecules which show decreased KF binding after nucleotide addition. Molecules which do or do not demonstrate extended dwells in the fingers-closed conformation immediately after nucleotide incorporation are indicated by groups b_bright_ and b_dark_, respectively.

DNA molecules were immobilised onto passivated glass surfaces as described previously.^27^ Surface-immobilised DNA molecules were imaged using a custom-built TIRF microscope^22^ and localised via the Cy5 dye attached to the primer strand, using a brief illumination (2–5 sec) with a red laser, which is turned off for the rest of the experiment; this initial illumination is evident as an excess signal in the DA channel during the first 2.4 s of our fluorescence time-series [e.g., Figures 1(B), 2)]. Observations of KF binding to the DNA molecules were performed in an imaging buffer consisting of variable concentrations of KF and nucleotide, in an imaging solution of 50 mM Tris, pH 7.5; 100 μg/ml BSA; 1 mM DTT; 5% glycerol; 1 mM Trolox (UV-treated); 1.9 mg/ml glucose oxidase; 0.075 mg/ml catalase; and 1.5% w/v D-glucose. Ultrapure nucleotides were purchased from Sigma Aldrich.

### Reaction initiation without fluidics

To observe the DNA polymerisation reaction in real-time, we used TIRF microscopy to first image and localise surface-immobilised extensible DNA molecules in a well containing imaging buffer without nucleotides. To initiate the DNA polymerisation reaction, we introduced a “reaction-initiating buffer” into the well via pipette. Defocusing during buffer addition was avoided by pipetting at liquid interfaces and using an autofocusing system to rapidly refocus the microscope [Continuous Reflective-Interface Feedback Focus System (CRIFF, ASI Imaging]. The autofocus system relied on a total-internally-reflected laser beam to infer focal plane position, which depended on solution refractive index (RI); when initiating the reaction, the mixed solutions needed to have matching RI for the CRIFF to function correctly. The use of pipette and autofocus system, rather than a fluidic chamber and syringe-pump system,^28^ provided a simple and robust experimental workflow and an excellent throughput.

We used two different techniques to initiate the reaction. In the first technique, KF was added to the well before the nucleotides. In this case, immobilised DNA molecules were localised via their attached fluorescent dyes in a well containing 20 µl of imaging buffer solution and 4 nM of KF. Data acquisition was started in order to image KF molecules binding and dissociating to the DNA; the average bound time for KF to the DNA template was ∼10 s.^27^ After ∼70 s, the reaction was initiated by pipetting 20 µl of a “reaction-initiating“ buffer into the well, which corresponded to the well imaging-buffer supplemented with 200 µM of dATP, dTTP, dCTP, and dGTP (Ultrapure Nucleotides, Sigma Aldrich). In this case, polymerisation could begin immediately at the DNA molecules which, at the time of dNTP addition, were already in complex with KF.

In the second technique, nucleotides and KF were added simultaneously to the well. In this case, DNA molecules were localised in a well containing 20 µl of imaging buffer solution, but in the absence of nucleotides or KF molecules. Approximately 10 sec after beginning data acquisition, DNA polymerisation was initiated by pipetting 20 µl of a reaction-initiating buffer containing imaging buffer, nucleotides at twice the desired concentration (which was variable), and 0.5–1 nM of KF. In contrast to the first method, DNA molecules were initially free of KF, and the lower concentration of KF produced a delay between the addition of buffer and polymerisation, which begun at each DNA molecule in a desynchronised manner, occurring upon binding of a KF molecule.

The observations of KF during the DNA polymerisation reaction were more challenging than our previous work,^27^ as the polymerisation reaction occurred rapidly (1–15 nt/s)^21^, ^22^, ^23^, ^24^, ^18^, ^25^ requiring continuous imaging during reaction initiation. Additionally, fewer events were observable, as only 1 field-of-view (FOV) could be observed per experiment (∼100 DNA molecules in a FOV), and the polymerisation reaction would only occur once for each DNA molecule in a field-of-view.

### Intensity trace extraction

Fluorescence time-traces at each DNA were extracted from TIRF movies using software described previously^27^ (Figure 1(B)). KF binding events to each DNA were identified by applying an intensity thresholding algorithm to each trace.^27^ To account for the variable illumination across a field-of-view (FOV), the thresholding value used was altered automatically for each DNA molecule, and set based on the median of observed intensity values between 400 and 3000 of the arbitrary units of the camera (AU). KF-binding events shorter than 0.5 s were discarded as they predominantly represented non-specifically bound KF-surface complexes.^27^ Contributions from KF-DNA complexes further than 0.5 pixels away from the initially localised DNA molecules were also removed. We calculated the apparent FRET efficiency using the ratio of donor and acceptor fluorescence emitted under donor excitation (DD/DA respectively) according to the equation E* = DA/(DA + DD).

### Hidden Markov modelling and polymerisation dwell extraction

Dwells in the fingers-open (E* ∼ 0.4) and fingers-closed conformations (E* ∼ 0.6) during KF-DNA binding events were extracted by fitting the first 5 sec of the KF-DNA binding events to Hidden Markov models (HMM) with 1–3 states to account for the two fingers conformational (and FRET) states and the bleached acceptor-dye state. The best fit was selected using maximum evidence criteria using previously documented software.^29^ Hidden Markov modelling to extract binary complex fingers-opening and closing dwells in the absence of polymerisation was performed as described previously.^27^

## Results

### Real-time observations DNA polymerisation at the single-molecule

In previous work, we characterised the conformation of KF in a binary complex with non-extensible (dideoxy-terminated) DNA substrates.^27^ Here, to observe the conformational status and conformational transitions of the KF fingers during real-time DNA synthesis, we used extensible template DNA constructs, allowing observation of the fingers’ conformations along the entire DNA polymerisation reaction. The template DNA constructs used (Figure 1(C)) were able to support the addition from 1 to 15 nucleotides depending on the set of nucleotides provided.

As in our previous work, we used TIRF microscopy to monitor the FRET efficiency of single fluorescently labelled KF molecules as they bind to surface-immobilised primer-template DNA molecules (Figure 1(A) and Methods), which were initially localised by an attached fluorescent dye (Cy5). Since polymerisation occurs rapidly (1–15 nt/s)^21^, ^22^, ^23^, ^24^, ^18^, ^25^ we added nucleotides during data acquisition, approximately midway through the experiment (see “KF added to well before nucleotides” in Methods).

To monitor the conformation of the fingers as KF copies up to 15 nt of template DNA, we initiated the reaction by adding a solution of dATP, dGTP, dCTP and dTTP to the well, to reach a final concentration of 100 µM (*Methods*). However, for the first ∼72 s of the experiment (Figure 1(B)), we observed KF binding to DNA in the absence of nucleotides, monitored via the fluorescence emitted at each DNA molecule position. As seen in a fluorescence time-trace at a single DNA molecule (Figure 1(B)), donor–acceptor labelled KF bound to and dissociated from the DNA molecules, as inferred via increases and decreases in fluorescence. Under donor excitation, fluorescence emission from the donor (“DD” fluorescence; green trace in Figure 1(B)) and the acceptor (“DA” fluorescence; red trace in Figure 1(B)) dye reported on KF binding to DNA, as well as on the fingers conformation (with the classification relying on the FRET efficiency signals). In the example of Figure 1(B), binding of a donor–acceptor-labelled KF molecule (KF_DA_) can be seen at ∼20 s and ∼60 s via the simultaneous DD and DA fluorescence increases, which correspond to significant FRET efficiency (E* > 0.2). Binding of a donor-only KF molecule (KF_D_) can also be seen at ∼45 s via the DD fluorescence increase; we note that our KF sample contains ∼30% of such donor-only molecules.^15^, ^27^

To study the KF-DNA binary complex formed on extensible template DNA molecules, we examined our timetrace segments prior to nucleotide addition (Figure 1, t < 72 s), and calculated FRET values (E*) for each frame during KF-DNA binding events. Prior to nucleotide addition (t ∼72 s), KF molecules that bound transiently to DNA interconverted between FRET values of E* ∼0.4 and E* ∼0.6; these represented transitions between the fingers-open conformation (E* ∼0.4) and fingers-closed conformation (E* ∼0.6), as we have published previously (Figure 1(D), lower panel, E* transitions between ∼62 s and ∼72 s).^27^ To measure fingers-opening and closing rates, we used Hidden Markov Modelling to extract dwells in the fingers-open and fingers-closed conformations. Histograms of these dwell times yielded the interconversion rates between the fingers-open and closed conformations for the KF-DNA binary complex (i.e., in the absence of nucleotides; Figure 1(E)). The fingers-closing rates of KF on extensible DNA substrates (k_closing_ = 1.4 ± 0.4 s^−1^) were comparable to those previously measured on dideoxy-modified primer-templates (k_closing_ = 1.1 ± 0.2 s^−1^). However, the fingers-opening rate on extensible templates was slower by a factor of ∼5 (k_opening_ = 5.3 ± 2 s^−1^) compared to that on non-extensible primers (k_opening_ = 29 ± 3 s^−1^), strongly suggesting that the presence of the 3′-OH stabilises the fingers-closed conformation.

Immediately upon nucleotide addition, those KF molecules already bound to DNA adopted a fingers-closed conformation and remained in an apparent closed conformation for a significant time, and until KF dissociated (Figure 1(D), lower panel, increase in E* from ~0.4 (open) to ~0.7 (closed) at ∼72 s). The overall extent of KF binding decreased soon after nucleotide addition (see example in Figure 1(B), where there is a sharp decrease in the frequency and duration of high fluorescence signals after ∼78 s). We interpreted the dwell in the fingers-closed state as the incorporation of multiple nucleotides onto the primer strand (which can be up to 15 additions, given the template DNA and the nucleotide set provided), as KF is known to enter the fingers-closed conformation in the presence of complementary nucleotides.^27^ Additionally, the dwells in the fingers-closed conformation were much longer than the equivalent dwells seen for the KF-DNA binary complex. The decrease in DNA binding shortly after nucleotide addition (i.e., after ∼78 s in Figure 1(B)) is consistent with the extension of the primer strand to form a DNA substrate with either a blunt end or a short single-stranded overhang (1–4 bp), both of which are well known to have a lower affinity for KF.^30^

### Visualisation of an ensemble of single DNA synthesis events

To analyse the polymerisation behaviour across our entire population of DNA molecules, we introduced a novel, concise and intuitive FRET timetrace representation for hundreds of single DNA substrate molecules from the same field of view. This visualisation approach preserved individual molecule behaviours, was minimally reliant on an external model, and allowed assessment of the whole data-set in an objective manner, validating that our analysis of individual single-molecule time traces represented the main behaviours of a population of molecules.

Our visualisation reduced the collection of ∼100 fluorescence time-series from each experiment (e.g., Figure 1(B)) into a 2D heat-map, with time on the x-axis, and a DNA index on the y-axis (Figure 2); each horizontal line represented the time-trace at an individual DNA molecule. The line was colour-coded at each time-point according to fluorescence intensity and FRET efficiency. Below a fluorescence intensity threshold, the line remains white, representing unbound DNA; for fluorescence above a threshold for more than 1 frame, a colour was used, representing the presence of KF. The colour-code used was based on the FRET efficiency at that time point: fingers-open conformations were coloured green (0.3 < E* < 0.53); fingers-closed conformations were coloured red (0.53 < E* < 0.8); KF molecules without an acceptor dye (due to either bleaching or incomplete labelling) were coloured blue (0 < E* < 0.3); and rare, unassigned high FRET values were coloured black (0.8 < E* < 1). This classification reduced the traces on single DNA molecules into a stack of 1D colour-coded lines (Figure 2). We then sorted these colour-coded lines to make the plots more readable: the lines were initially sorted by the extent of KF-DNA binding *before* nucleotide addition (Figure 2, t < 72 s); since 32% of immobilised DNAs showed low or no KF binding (SI Figure 1, group c), they were removed from further analysis. Thereafter, we sorted the remaining lines by the extent of KF-DNA binding *after* nucleotide addition (Figure 2, t > 72 s).

Our visualisation demonstrated that the ensemble of KF molecules exhibited behaviours broadly consistent with the time-trace in Figure 1. As expected, before nucleotide addition, KF-DNA complexes were predominantly in the fingers-open conformation (Figure 2; events between 0 and 72 s are 82% green, 18% red). Upon nucleotide addition, the number of polymerases in the fingers-closed state (red) increased significantly, from ∼5 to ∼22 (out of ∼44), with these molecules remaining closed for a few seconds, which we interpreted as KF being in the process of DNA polymerisation (Figure 2; binding events between 72 and 90 s are predominantly red, indicating fingers-closed KF). Nucleotide addition induced a gradual decrease in the number of KF-DNA complexes, such that by the final frame only ∼10 complexes remained bound, down from ∼45 complexes prior to nucleotide addition (Figure 2, decreased binding between 90 and 160 s). We interpreted this as the effect of the formation of DNA blunt-ends and short overhangs.

We further grouped DNA molecules via their behaviours: we identified “persistent binders” as the ∼19% of DNA molecules which did not demonstrate clear decrease in KF binding by the end of the experiment (Figure 2, group a: DNA molecules 108–132). This group also did not exhibit clear changes in conformational state upon nucleotide addition. We attribute this behaviour to persistent non-specific adsorption of KF to the surface, possibly caused by defects in the PEG passivation.

We also identified a group of DNAs with decreased binding after polymerisation, which corresponded to the ∼81% of DNAs which showed significant KF binding before nucleotide addition, followed by decreased KF binding by the end of the experiment, consistent with DNA polymerisation (Figure 2, group b, DNA molecules 1–107). Within group B, ~61% of molecules (“the bright B subgroup”) featured significant levels of binding in the first few seconds after nucleotide addition, before showing decreasing binding (Figure 2, DNAs 42–107). Most of these binding events featured KF in the fingers-closed (red) conformation – either with KF being bound before nucleotide addition, and transitioning to the closed state, or with KF binding and then entering the closed conformation. We interpreted this as a KF polymerising the DNA in the fingers-closed conformation, and generating an extended DNA strand with lower KF binding affinity.^30^

The remaining ~39% of group b (“the dark B subgroup”) exhibited typical KF binding initially, and decreased KF binding by the end of the experiment, suggesting that DNA extension had occurred (DNAs 1–42). However, there were no clear KF binding events after nucleotide addition at these molecules. We attributed these to “dark polymerisations“, which correspond to DNA extensions performed by unlabelled or acceptor-only labelled KF molecules, as the fraction of these events (~39%) was consistent with the fraction of unlabelled and acceptor-only polymerase in our KF sample [~25% and ~30%, respectively^15^, ^27^].

### KF fingers are predominantly closed during polymerisation at high dNTP concentrations

To validate our result that addition of high dNTP concentration and subsequent DNA synthesis caused KF to adopt primarily the fingers-closed conformation, we used a different approach, wherein 100 μΜ of dNTPs and a lower concentration of KF (here, 0.5 nM vs. 4 nM in the previous section) were added to a glass slide containing only immobilised DNA templates. The low concentration of KF ensured the presence of a stochastic time delay between the addition of KF to the slide (due to the time required for KF to diffuse and bind to DNA), and its subsequent binding to DNA, which in turn allowed mixing and equilibration of the system before DNA polymerisation began. The solution of KF and dNTPs was added ∼10 s after beginning data acquisition (see “nucleotides and KF added simultaneously” in Methods), and led to most traces showing a stochastic time-delay until a KF molecule bound to a DNA molecule, followed by near-immediate (within our temporal resolution of 40 ms) adoption of the fingers-closed conformation during polymerisation (red stripes in SI Figure 2).

FRET efficiency histograms of the KF-DNA binding events at individual DNAs demonstrated that the DNA-bound KF remained predominantly in the fingers-closed conformation at ∼71% of DNA molecules (see heatmap for DNAs from 1 to 100 in SI Figure 3A). KF binding at the remaining DNA molecules was either predominantly donor-only, or in the open conformation, or had aberrant FRET values (DNAs from 101 to 141 in SI Figure 3A); we attributed the presence of aberrant FRET values or open conformation KF to the population of KF non-specifically bound to the surface (group A in Figure 2). For the DNA molecules exhibiting clear polymerisation, KF molecules spent ∼95% of their time in the fingers-closed conformation (SI Figure 3B, FRET histogram of all KF molecules binding to DNAs 1–100).

Taken together, our results clearly establish that KF molecules, upon binding template DNA, rapidly enter the fingers-closed conformation and remain primarily closed until completing polymerisation.

### DNA polymerisation by 5, 10 or 15 nucleotides

We then investigated the kinetics of processive DNA polymerisation by taking advantage of the sequence of our primer-template DNA, which permitted primer extension by a different number of bases depending on the dNTP subset used (Figure 1(C)). Primer extension of up to 5 bases could be achieved by using a dATP and dTTP subset; similarly, extension of up to 10 bases could be achieved using a dATP, dTTP and dGTP subset. Finally, full extension of the primer by 15 bases could occur when all nucleotides were added. Our experiments began in the absence of KF, with KF and dNTPs being added together to initiate the reaction.

While using very low concentrations of dATP and dTTP (0.25 µM; a low concentration relative to a K_M_ of 1.4–3.6 μM of KF for complementary nucleotide incorporation^31^, ^32^) to permit extension by only 5 bases, we observed KF binding events that exhibited a small number of rapid oscillations between the two main fingers conformations, before returning to the fingers-open conformation, and dissociating (SI Figure 4; we interpret this behaviour (which is similar to the one seen in the absence of dNTPs) as fingers dwells in the open state while KF is waiting for nucleotides to bind before incorporation.

**Figure 3:**
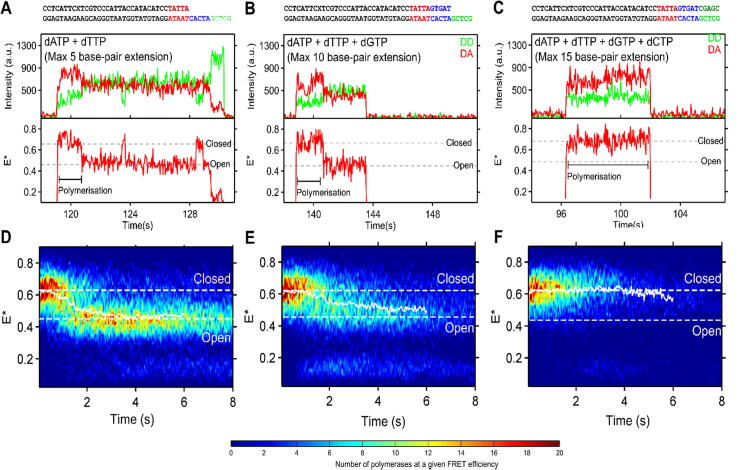
(A–C) Fluorescence time-traces demonstrating KF binding to DNA in the presence of different nucleotide subsets. Donor-excitation donor-emission (DD – green); Donor-excitation, acceptor-emission (DA – red); FRET efficiency (E* – red). (A) A + T nucleotides (max 5-nt extension). KF-DNA binding at ∼119 s shows KF initially closed, interpreted as polymerisation, and subsequent fingers-opening at ∼121 s, remaining predominantly open before dissociating. Binary complex dynamics seen at ∼123.5 s and ∼129 s. (B) A + T + G nucleotides (max 10-nt extension). KF binding at ∼138 s shows KF initially closed during polymerisation and then opening at ∼141 s, remaining open before dissociating. (C) A + T + G + C nucleotides (max 15-nt extension). KF binding at ∼96 s shows KF initially closed whilst polymerising and dissociating at ∼102 s. (D–F) Time-series histograms of the FRET efficiency of KF binding events, post-synchronised across an entire experiment and represented in a colormap. Molecules which begin in the fingers-closed conformation are selected (the remainder are seen in SI Figure 5). Results are collated from KF binding events to 279, 306 and 236 DNA molecules for panels D, E, and F, respectively. (D) A + T nucleotides (max 5-nt extension): KF molecules that begin in the closed conformation returning to the open conformation by ∼2 s. (E) A + T + G nucleotides (max 10-nt extension): KF molecules that begin in the closed conformation return to the open conformation by 2–3 s. (F) A + T + C + G nucleotides (max 15-nt extension): KF molecules that begin in the closed conformation do not return to the open conformation, instead dissociate by ∼4 s. Contours are used to aid visualisation; in reality, the data are quantised in 40-ms steps.

To minimise any dwells required for nucleotide to bind, and to focus on the kinetics of processive polymerisation after nucleotide binding, we ran experiments using a much higher concentration of nucleotides (100 μM, much higher than the aforementioned K_M_ values). Upon adding dATP and dTTP at 100 μΜ (Figure 3(A)) to permit a 5-nt primer extension, KF entered the fingers-closed conformation rapidly upon binding to DNA, (<40 ms; see binding event at ∼119 s, Figure 3(A)), remained closed for a period of time (∼1.5 s in Figure 3(A)), and then returned to the fingers-open conformation (after ∼121 s in Figure 3(A)), with brief excursions to the fingers-closed state (at ∼124 s at Figure 3(A)). We interpreted this profile of transitions as a 5-nt extension of the DNA by KF, followed by binary complex dynamics, since the 5-nt extensions would leave a 10-nt ssDNA overhang, capable of retaining KF for a few more seconds.

Upon adding dATP, dTTP and dGTP at 100 μΜ (Figure 3(B)) to permit a 10-nt primer extension, the profile of transitions was similar to the 5-nt extension (e.g., see KF binding event at ∼138 s in the closed conformation, opening after ∼2 s, and staying in the open state for ∼3 s more before KF dissociation from the DNA).

Upon adding the full dNTP set at 100 μΜ (Figure 3(C)) to permit a full 15-nt primer extension, KF again appeared closed upon binding, remained closed for a period, but then dissociated without clearly returning to the fingers-open state. We interpreted this as full extension of the primer, to the point where the reduced ssDNA overhang would substantially reduce the DNA-binding affinity of KF, leading to rapid KF dissociation.

Since the stable return of the KF to the open state signifies the completion of polymerisation in the partial-extension experiments, we reasoned that the first fingers-closed dwell in the presence of 100 μM dATP and dTTP, and in the presence of 100 μM dATP, dTTP and dGTP, corresponds to the time taken to polymerise DNA by 5 or 10 nt, respectively. During the entire polymerization time, KF appears closed and does not detectably open until completion or dissociation, suggesting that any dwells at the open state are short compared to our exposure time of 40 ms.

This general behaviour is also reflected in time-series FRET histograms produced by post-synchronising KF-DNA binding events (Figure 3(D–F)). We filtered for DNA molecules in which KF molecules start their dwell in the apparent fingers-closed conformation, to remove the population of “persistent binders” that show no dNTP specific activity (Figure 2, group A). The histograms of post-synchronised KF-DNA binding events show that the ensemble of KF molecules follow the same pattern as seen for the single-molecule traces; for the 5-nt and 10-nt extensions, histograms begin at high FRET (E* ∼ 0.65) and return to low FRET (E* ∼ 0.45) over time (Figure 3(D–E)), whilst for the extension of up to 15 nt, the histogram begin and stays at high FRET, with only the number of events decreasing over time as polymerases dissociate after copying all or nearly all of the DNA template (Figure 3(F)). The removed molecules, which do not begin in the finger-closed conformation, are a minority population; do not show similar dynamical behaviour; and have a strong signal for acceptor-only labelled molecules (SI Figure 5).

### Kinetics of DNA polymerisation

**Figure 4:**
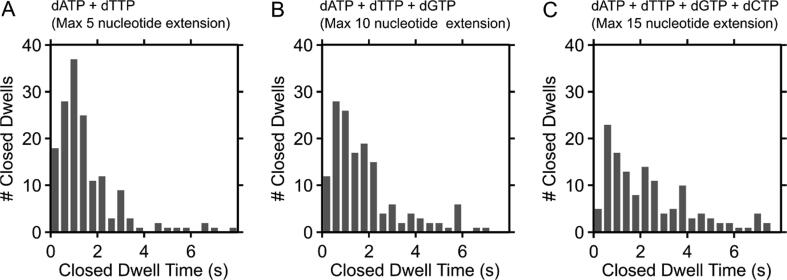
First dwells in the fingers-closed conformation upon binding of KF to DNA in the presence of A + T, A + T + G, and A + T + G + C, allowing maximal extension by 5, 10 and 15 nucleotides, respectively (panels A, B, C). Dwells reflect the time taken to polymerise the DNA and are extracted using HMM from the first dwell in the KF fingers-closed conformation. Distributions are seen to broaden on increasing incorporation lengths. Results are collated from KF binding events to 279, 306 and 236 DNA molecules for 5-, 10-, and 15-nt extensions, respectively.

To measure the time taken to polymerise 5, 10 and up to 15 nucleotides during primer extension, we extracted the first dwell in the fingers-closed conformation (interpreted as the apparent DNA polymerisation time, hereafter “polymerisation time”) upon binding of KF to DNA in the presence of nucleotides (see examples of HMM fits in SI Figure 6). We then plotted histograms of the polymerisation time at each DNA under conditions permitting 5, 10 and up to 15-nt extension (Figure 4).

The histogram of polymerisation times for the 5- and 10-nt extensions show peaked distributions, with the majority of events and the distribution peaks appearing between 0.5 and 2 s, and tails extending to longer times, which become more pronounced for the 10-nt extensions. This characteristic shape of distributions points to the presence of several sequential rate-limiting steps, as expected from the presence of multiple cycles of nucleotide addition for a single KF-binding event.^33^, ^34^, ^35^ There was not a large shift in the polymerisation times between 5-nt and 10-nt extensions, with the mean polymerisation time only increasing from 1.9 s for 5-nt extensions to 2.2 s for 10-nt extensions. This suggested that the bases between the 5–10 positions may be incorporated faster that the bases between positions 1–5. The dwell times in the presence of all nucleotides (Figure 4(C)) are more difficult to interpret, as it is unclear whether the polymerase extends the DNA primer fully or leaves short (∼3-nt) overhangs before dissociating; however, the mean time for a maximal 15-nt extension is 2.8 s, strongly suggesting that the reaction proceeds beyond the extension of 10-nt.

To explore further the distribution of polymerisation times, we simulated the dwells in the fingers-closed conformation for extension by 5, 10 or 15 bases. The length of each dwell was simulated via summing 5, 10 or 15 exponentially-distributed random variables; after repeating 10,000 times for each extension case, we create simulated versions of the distributions in Figure 4.

When assigning each base an identical incorporation time, based on the measured incorporation time of a single base (3.2 s^−1^, see next section), the polymerisation time for the 5-nt extension fit reasonably well to measured values (SI Figure 7(A); compare simulation [blue line] with measurements [grey bars]). However, the simulated 10- and 15-nt polymerisation-time distributions diverged from the measurements, significantly overestimating the mean polymerisation times (SI Figure 7(B, C)) and suggesting there must be incorporations faster than 3.2 s^−1^. Furthermore, the central limit theorem states that the sum of independent, identically distributed random variables will approach a Gaussian distribution as the number of variables increases; instead, the polymerisation-time distributions clearly deviated from a Gaussian, suggesting that modelling nucleotide incorporations as independent, identically distributed variables is not appropriate – and instead pointing to the presence of different incorporation rates at each base position. The broad distribution for the 15-base extension case suggests a combination of fast and slow steps.

We then modelled the incorporation time as identical for each base, but allowed the exponential parameter to freely fit the data. This extracted incorporation rates of 4, 5.8 and 5 s^−1^ for the 5, 10 and 15-base extension cases, respectively (SI Figure 8), but the fits did not match the measured data. Finally, we fit the data to a model in which each base-type (A, T, C or G) had a unique incorporation rate. This treatment also did not provide good fits to the data, suggesting there are kinetic processes at play besides differing incorporation rates for each base, such as a heterogeneity in polymerisation rates; these processes may be caused by pausing, sequence dependence, or DNA secondary structure.

To control for the contribution of KF dissociation or photobleaching mid-polymerisation, the data were reanalysed using only molecules which return to the fingers-open state after polymerization. For the 5- and 10-nt extensions, the form of the dwell-time distributions was not significantly altered (SI Figure 9), indicating that photobleaching or early dissociation do not alter our conclusions. For the 15-nt case, few events remained after filtering, indicating that KF dissociates rapidly after primer extension.

### Kinetics of single-nucleotide incorporation

**Figure 5:**
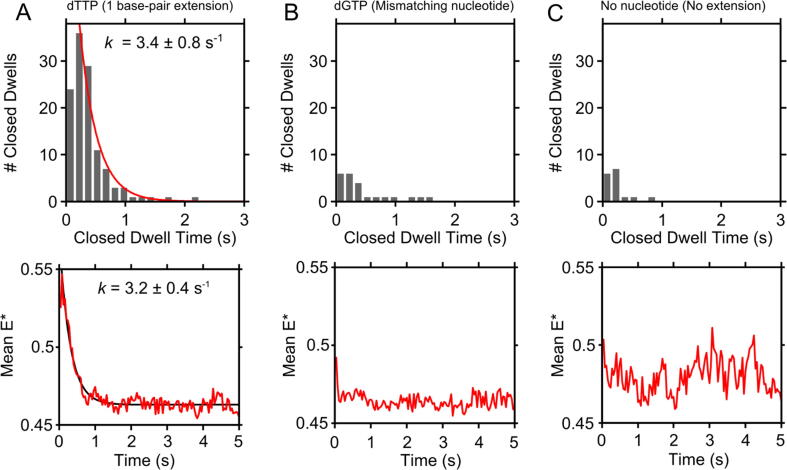
The kinetics of single-nt extension events by KF. Results are collated from KF binding events to 453, 568 and 211 DNA molecules for panels A, B and C, respectively. (A) Top: durations of the first fingers-closed dwell at KF-DNA binding events in the presence of nucleotide complementary to the first template base. Fit to a single-exponential extracts the rate of dTTP incorporation at 3.4 ± 0.8 s^−1^. Bottom: mean E* of post-synchronised KF-DNA binding events across a collection of DNAs in the presence of (A) complementary nucleotide (dTTP) demonstrating an exponential decrease in mean E*. Polymerisation in the presence of dTTP is evidenced by the much higher number of events compared with no nucleotides, or mismatching nucleotide, in panels B, C, respectively. (B) As for panel A, but for a nucleotide (dGTP) non-complementary to the first template base. No change in mean E* is observed. (C) As for panel A, but in the absence of a nucleotide. No change in mean E* is observed.

We then turned our attention to measurements of a single nucleotide incorporation onto the DNA; this was achieved by initiating polymerisation in the presence of dTTP alone at 100 μM. As above, we observed fingers-closed dwells upon binding of KF to DNA, but these dwells were shorter than during 5- and 10-nt extensions, consistent with fewer incorporations. After extracting the fingers-closed dwells using HMM (SI Figure 10), we obtained a dwell-time histogram which we fit to single-exponential kinetics, suggesting a single rate-limiting step with a rate of 3.4 ± 0.8 s^−1^ for the addition of a single nucleotide (Figure 5(A), upper panel; note that the fit does not match well to the first bin, likely due to the systematic undercounting of single- and dual-frame events, due to our exposure time of 40 ms). To establish that the dwells we observe correspond to specific dNTP binding and incorporation, we repeated this experiment with a non-complementary nucleotide, as well as without nucleotides; in both cases, we observed far fewer closed state dwells (∼90% fewer events; Figure 5(B–C), upper panels), supporting our interpretation that we are indeed measuring the kinetics of single-nucleotide incorporations.

To verify our results based on HMM analysis, we also extracted the rate of single-nucleotide incorporation after post-synchronising the KF-DNA binding events and calculating a mean FRET (E*) trace based on the synchronised events (Figure 5(A), lower panel). For dTTP incorporation, the collection of KF molecules initially demonstrated a high-FRET efficiency, followed by a decrease in mean E* at a rate of 3.2 ± 0.4 s^−1^, as KF molecules returned to their fingers-open conformation after completing polymerisation in the fingers-closed state (Figure 5(A), lower left panel, showing a mean E* ∼0.55 at t = 0, reducing to E* ∼0.46 by t = 1). In contrast, experiments using either non-complementary nucleotides or no nucleotides show negligible change in their mean FRET efficiency over time (Figure 5(B–C), lower panels; mean E* remains at 0.46–0.47).

We also repeated this experiment using modified primer-template DNAs in which the first templating base was changed to either a T, a G or a C, in order to measure the polymerisation rates of single dATP, dCTP or dGTP nucleotides separately. These exhibited similar but slightly slower rates, which were extracted using the post-synchronised mean-E* traces (2 ± 0.4 s^−1^, 1 ± 0.2 s^−1^, and 1.5 ± 0.4 s^−1^, respectively; see SI Figure 11).

## Discussion

Here, we present a method which allows extended real-time observations of the conformation of KF fingers during the actual DNA polymerisation reaction on immobilised DNA templates; the DNA sequences on the templates allowed us to explore the sequence-dependence and length-dependence of the kinetics of fingers conformational changes. Historically it has been challenging to dissect the order and measure the rate constants for the post-chemistry reaction steps due to the need to synchronise the system for ensemble kinetic experiments. Our single-molecule assay provides a direct and robust method to characterise post-chemistry steps in more detail by removing the need for synchronisation. Our analysis was helped by an intuitive data visualisation technique that presents hundreds of single-molecule behaviours simultaneously.

### Fingers-opening occurs after the slow post-chemistry step of the nucleotide addition cycle

During DNA polymerisation at high nucleotide concentrations, we observed that KF adopts predominantly the fingers-closed conformation; any detectable transitions to the fingers-open conformation are minimal at our 40-ms time resolution. To place these observations in the context of the nucleotide addition cycle, we note that the cycle begins with nucleotide binding to the KF fingers-open conformation, followed by fingers-closing (∼140 s^−1^),^3^, ^6^ binding of a second Mg^2+^ ion, a pre-chemistry rate-limiting step, and chemical incorporation of the nucleotide onto the primer (∼40–87 s^−1^ for all steps up to [and including] chemical incorporation).^3^, ^6^, ^18^, ^36^, ^37^ Following chemical incorporation, there is a slow post-chemistry step (15 s^−1^) which is ascribed to a non-covalent transition, followed by pyrophosphate release.^18^ Translocation and fingers-opening also occur after chemistry, although their place in the sequence of events is unclear.^38^

Given that fingers-closing (140 s^−1^) is faster than our time-resolution (25 s^−1^ frame rate), we did not expect to observe dwells in the fingers-open conformation during the early reaction stages. Indeed, we observed very few fingers-open dwells during polymerisation, suggesting that additionally, any post-chemistry steps in the fingers-open conformation also occur faster than 25 s^−1^.

**Figure 6:**
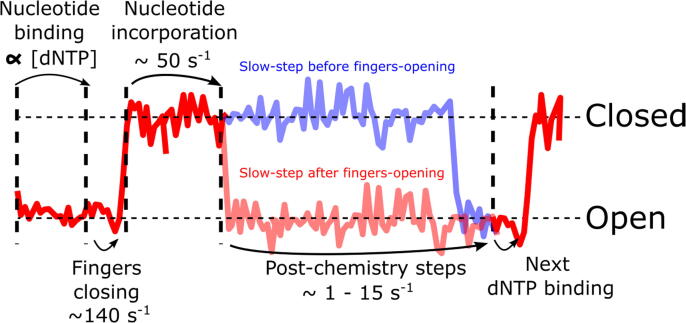
Schematic FRET time-series illustrating KF’s conformation overlaid with the known reaction scheme and associated time-scales. A single nucleotide incorporation cycle is seen between the left-most and the right-most vertical dashed lines. Nucleotide binding occurs in the fingers-open conformation, whose rate is proportional to nucleotide concentration, allowing us to minimize this dwell in our experiments. After nucleotide binding, fingers close rapidly, and nucleotide incorporation occurs in the closed conformation. Thereafter, the conformation of the KF fingers was previously unknown, and the blue and red lines show alternative possibilities in which the fingers open before (red) or after (blue) the post-chemical slow step. Note that our time resolution of 40 ms precludes direct observation of open-state dwells during finger-closing at high nucleotide concentrations.

The fingers-closed dwells thus act as a proxy measurement for the time taken to complete processive polymerisation by a single DNA polymerase molecule, as the fingers-open dwells are shorter than our time-resolution (40 ms). Given processive polymerisation is rate-limited by the post-chemistry slow step, this step must therefore occur while KF is in the fingers-closed conformation, i.e., fingers-opening occurs *after* the rate-limiting post-chemistry step. If fingers-opening were to occur *before* the slow post-chemistry step, we would observe a prolonged dwell in the fingers-open conformation after chemistry, until the slow post-chemical step was completed, allowing KF to close around the next incoming nucleotide (red schematic FRET trace, Figure 6). Instead, we observe long dwells in the fingers-closed conformation, with no apparent open-state dwells between incorporations (blue FRET trace, Figure 6). Although we indicate open-state dwells in Figure 6, these dwells are rapid at high dNTP concentrations, and are not observed with our time resolution of 40 ms, except for low dNTP concentration (as in SI Figure 4) which results in long open-state dwells as the nucleotides bind less frequently.

Fingers-closed dwells during single dTTP incorporations are also much longer (3.4 ± 0.8 s^−1^) than would be expected on the basis of the rate of single-nucleotide incorporation alone (∼40–87 s^−1^).^3^, ^6^, ^18^, ^36^, ^37^ This supports our conclusion that fingers-opening after chemistry must be defined by the slow-step *after* chemistry, as the steps before chemistry are too rapid.

### Processive polymerisation is likely to be rate-limited by pyrophosphate release and fingers opening

Our observations provide insight on the nature of the slow post-chemistry step. Since the rate-limiting event occurs before finger opening, one possible option is that the slow step is the fingers-opening step itself. A second option is that post-chemistry fingers-opening is fast, but it is rate-limited by a preceding event, such as pyrophosphate release. This second option is consistent with a model based on high-resolution structures of complexes of T7 RNA polymerase, a single-subunit RNAP with structural and mechanistic similarities of family A DNA polymerases; the model proposed that release of pyrophosphate facilitates fingers opening and subsequent translocation.^39^ Molecular dynamics simulations of the BSt DNA polymerase based on the T7 RNA polymerase structures^40^ also supported the model mentioned above, showing that fingers-opening from a post-chemistry conformation occurs more easily in the absence of PPi.

Our results show that the rate of single-nucleotide polymerisation based on post-chemistry fingers opening in the presence of PPi is 3.4 ± 0.8 s^−1^. This rate is significantly slower to that of opening rate in the binary complex (i.e., in the absence of PPi; 5.3 ± 0.8 s^−1^), suggesting that PPi release could contribute to the post-chemistry rate-limiting step of the transition. This two-step process could account for the apparent biexponential form of our single-nucleotide incorporation dwells, although further experiments would be required to confirm this (to account for any time-resolution artifacts).

However, even in the absence of pyrophosphate, fingers-opening is clearly a slow transition, as the results for fingers-opening in the binary complex indicate. The exact structure of the pre-chemistry fingers-closed conformation in the binary complex is unknown, but could either represent KF with its fingers closed around the absent base at the insertion site, or, more likely, KF translocated backwards and closed around the previous base pair; we find this hypothesis to be more likely, since the product of the previous reaction cycle resembles the catalytic substrate desired by the polymerase, and since backward and forward translocation in the absence of nucleotides is expected to be thermally driven and fast, as has been estimated in studies supporting thermal-ratchet models for other high-fidelity DNA polymerases.^41^ In essence, the binary-complex opening could be performing some of the final steps of the nucleotide addition cycle, which involve fingers-opening from the product but in the absence of pyrophosphate.

In support of our hypothesis that the binary complex is interacting with the previous base-pair, we have observed that the binary complex fingers-opening rate decreases 5.5-fold when using dideoxy-terminated DNA primers instead of extensible primers, suggesting that the fingers interact with the 3′-OH in the binary complex fingers-closed conformation (an effect that could require backward translocation). More specifically, the fingers-opening step in the absence and presence of 3′-OH in the binary complex occurred at rates of k_open_ = 29 ± 3 s^−1^(Ref. 27) and k_open-OH_ = 5.3 ± 0.8 s^−1^, respectively. Using Arrhenius kinetics, we obtain an energetic change of $\frac{k_{1}}{k_{2}}=\frac{e^{-\frac{E}{K_{B}T}}}{e^{-\frac{E+5}{K_{B}T}}}=e^{\frac{5}{K_{B}T}}$of ∼1 kcal/mol due to this 3′-OH interaction, which is on the order of a hydrogen bond (1–3 Kcal/mol).

In summary, the similarity in fingers-opening rates of the binary complex and the post-incorporation complex, are suggestive of a model in which PPi release and fingers-opening constitute the main rate-limiting post-chemistry steps. The rate-limiting step could also be due to some other uncharacterized process before fingers-opening, but the slow rate of fingers-opening from the binary complex indicates that any slow step would be included within a set of steps associated with fingers-opening. Fingers-opening and PPi release steps are very likely to occur before the translocation step and before the next template nucleotide inserts into the pre-insertion site, as previously suggested^39^, ^40^; in any case, regardless of its place on the cycle, translocation is expected to be fast and not rate-limiting.^42^

Our approach can be extended to characterise the fingers-opening and closing kinetics in the presence of different nucleotide derivatives, DNA substrates, and DNA polymerases, and informing on the energy landscape and mechanisms of their polymerisation reactions.

### Heterogeneity in incorporation rates

We also used the KF dwell times in the closed-fingers state to estimate rates of DNA polymerisation by single DNA polymerases on single DNA substrates. The fingers-closed dwells during the 5- and 10-nt extensions provided polymerisation rates of 2.6 nt/s and 4.5 nt/s respectively, which are within the broad range of 1–15 nt/s previously measured for processive DNA polymerisation at room temperature and for 10–100 μM dNTPs [4.5 and 6 nt/s for processive DNA polymerisation of ssDNA,^21^, ^22^ 1.2 nt/s and 14 nt/s for strand displacement synthesis,^23^, ^24^ and 15 nt/s and 1.2 nt/s for processive single-nucleotide incorporations^18^, ^25^]. Our measured rates are expected to be somewhat different to bulk extension rates, as our relatively short extensions will not average over heterogeneity caused by surrounding sequence, variation in the rate of incorporated nucleotides, secondary structure, or any pause sites.

Notably, the mean time taken to polymerise 10 nt (2.2 s) was only slightly longer than the mean time taken to polymerise 5 nt (1.9 s), suggesting that bases 6–10 (GTGAT) were incorporated much more rapidly than bases 1–5 (TATTA). Our measurements (SI Figure 11), along with published work,^6^, ^25^ suggest that this large difference cannot be explained simply by the variation in the incorporation rates of individual nucleotides.

Another possible cause of heterogeneity in the processive incorporation rate is pausing, previously observed and attributed to hairpins or secondary structure; Pyr-GC motifs; or trinucleotide repeats. However, these features are absent in our DNA sequence, and our template overhang has no secondary structure at room temperature.

This large variability in the apparent speeds of processive polymerisation could be attributed to sequence dependence; whether this dependence is predominantly due to the single-stranded or double-stranded regions of the DNA substrate is currently unclear, and future work could address this by measuring polymerisation rates on a variety of DNA sequences. The large number of potential sequence combinations would make an exhaustive characterisation challenging; however, this challenge may be overcome by a linkage between polymerisation kinetics and DNA-sequencing on the same instrument.^43^, ^44^

## CRediT authorship contribution statement

**Geraint W. Evans:** Investigation, Methodology, Formal analysis, Writing – original draft. **Timothy Craggs:** Resources, Writing – review & editing. **Achillefs N. Kapanidis:** Conceptualization, Supervision, Funding acquisition, Writing – review & editing.
