# Supplementary material for: The Rate-limiting Step of DNA Synthesis by DNA Polymerase Occurs in the Fingers-closed Conformation
Source: J Mol Biol. 2022 Jan 30;434(2):167410. doi: 10.1016/j.jmb.2021.167410 (PMC8783057; doi:10.1016/j.jmb.2021.167410)
Supplement: Supplementary data 1 [file mmc1.docx]

**SUPPLEMENTARY INFORMATION**


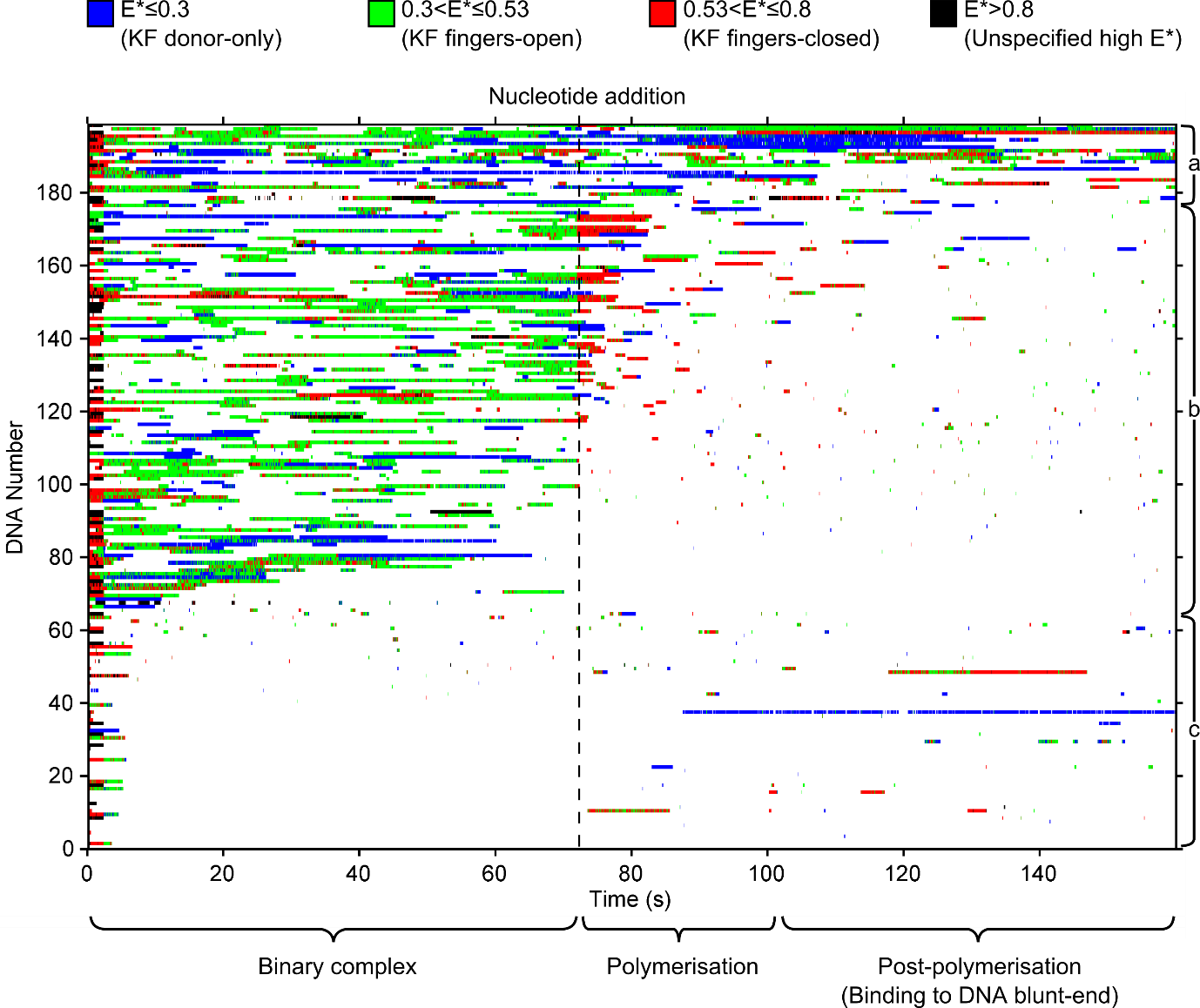


**SI Figure 1**. Visualisation representing the binding and conformation of KF molecules to

an ensemble of ~ 200 DNA molecules. Each horizontal line represents fluorescence time-series

at a DNA molecule (as in Fig 1B). Fluorescence increases caused by KF binding events are represented in colour, with the colour reflecting the FRET efficiency at each time point, as indicated in the figure. These FRET values correspond to the following states: Green: KF fingers-open. Red: KF fingers-closed. Blue: Donor-only KF. Black: Unspecified high FRET. Binding events before nucleotide addition (dotted line) are seen to be predominantly open (green), consistent with binary complex behaviour. A large increase in the number of fingers-closed KF molecules (red) is seen after nucleotide addition, which we interpret as KF molecules polymerising DNA. Decreased KF-DNA binding is seen subsequent to these polymerisations. The period between 0-3 seconds is due to the activated red laser used to localise the DNAs. Group (a): DNA molecules which do not show decreased KF binding after nucleotide addition. Group (b): DNA molecules which show decreased KF binding after nucleotide addition. Group (c): DNA molecules which show little KF binding at all. These molecules and were attributed to low intensity DNA molecules located on the edge of the FOV, or to the mistaken identification of background fluorescence as DNA molecules. The remaining DNA molecules were sorted by extent of KF binding after nucleotide addition.


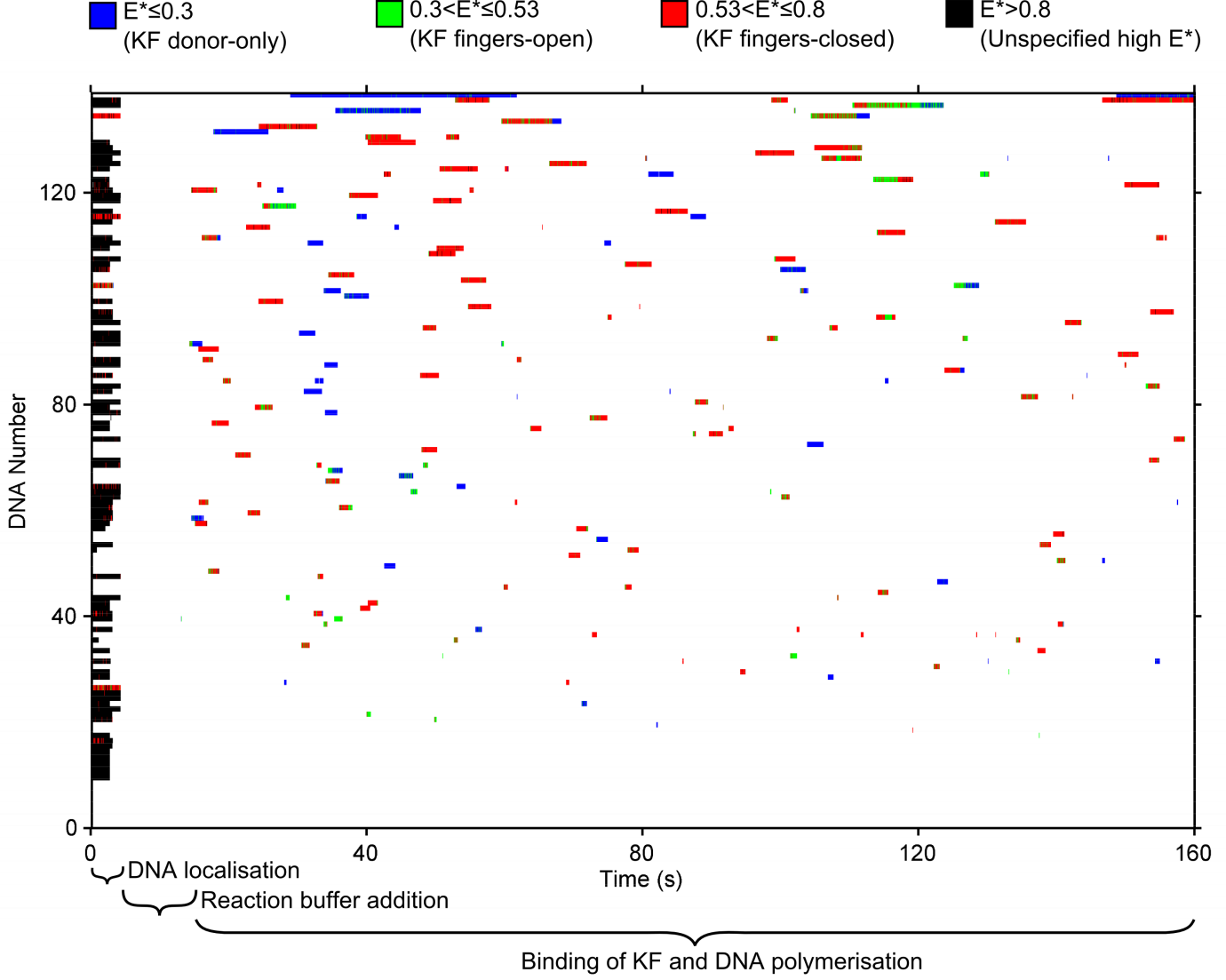


**SI Figure 2**. Visualisation representing the binding and fingers-conformation of KF molecules binding to an ensemble of DNA molecules. Each horizontal line represents a fluorescence time-trace at a DNA molecule, with KF binding events represented in colour, the colour representing the E* at each time point. Green: KF fingers-open. Red: KF fingers-closed. Blue: Donor-only KF. Black: Unspecified high FRET. The period between 0-5 s is due to the DNAs being localised when the red laser is switched on. KF and all nucleotides at 100 µM are added at ~10 s, after which KF binding events are distributed in time due to the stochastic binding as KF diffuses to binds the DNA. KF is seen to be predominantly fingers-closed, consistent with the involvement of KF in polymerising DNA.


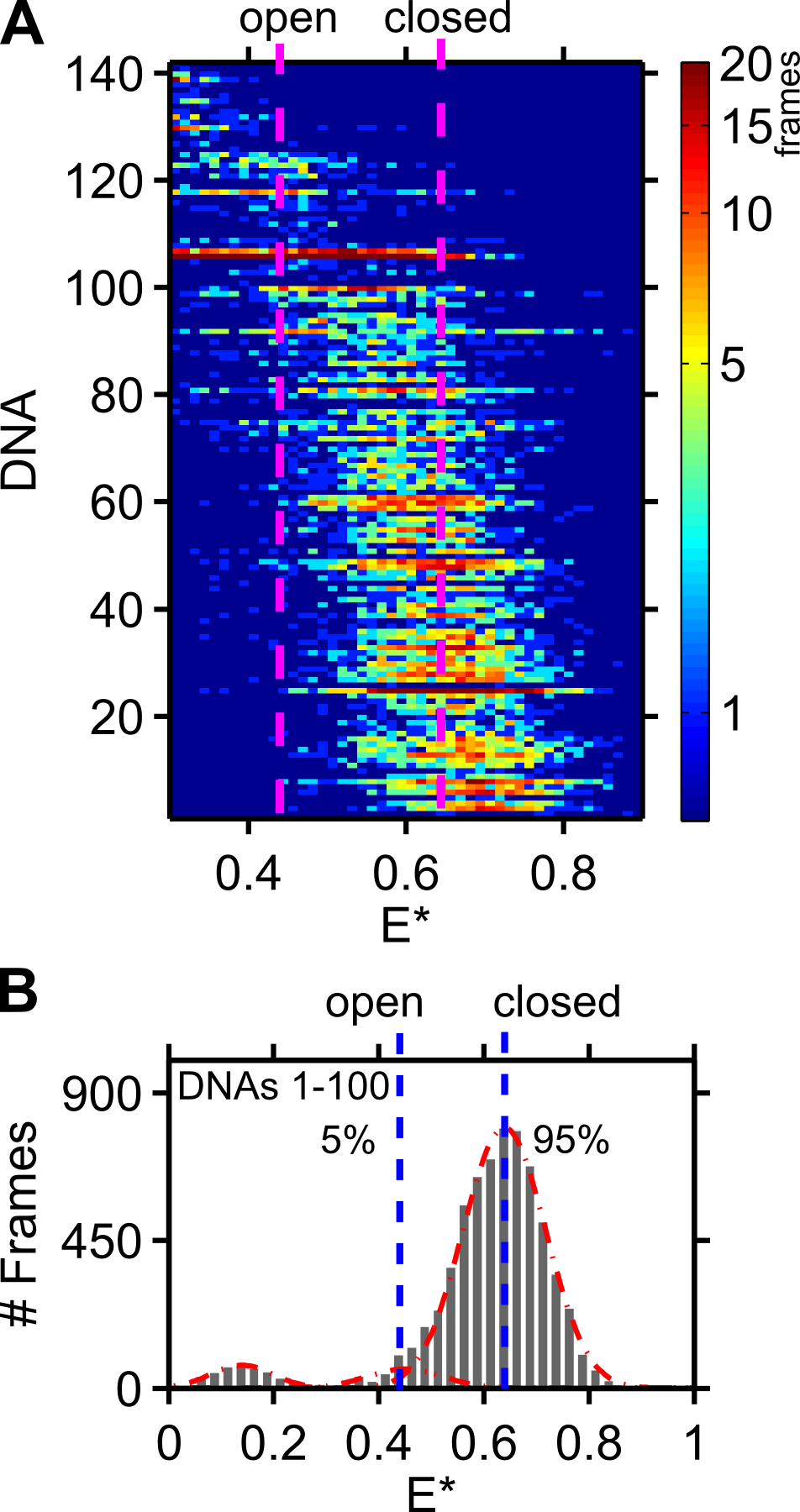


**SI Figure 3**. (A) FRET efficiency histograms of KF binding events at each DNA, represented in a colormap. These histograms were and stacked and sorted via mean FRET. The colour bar represents number of frames spent at each E* value. KF binding at DNA molecules 1-100 behave in a similar manner and are predominantly fingers-closed. Binding at DNAs (101-141) are donor only, fingers-open, or aberrant.

(B) Summed histograms of FRET efficiencies at DNA molecules 1-100, demonstrate KF to be 5% fingers-open and 95% fingers-closed. (Low FRET peak from donor-only molecules.)

*
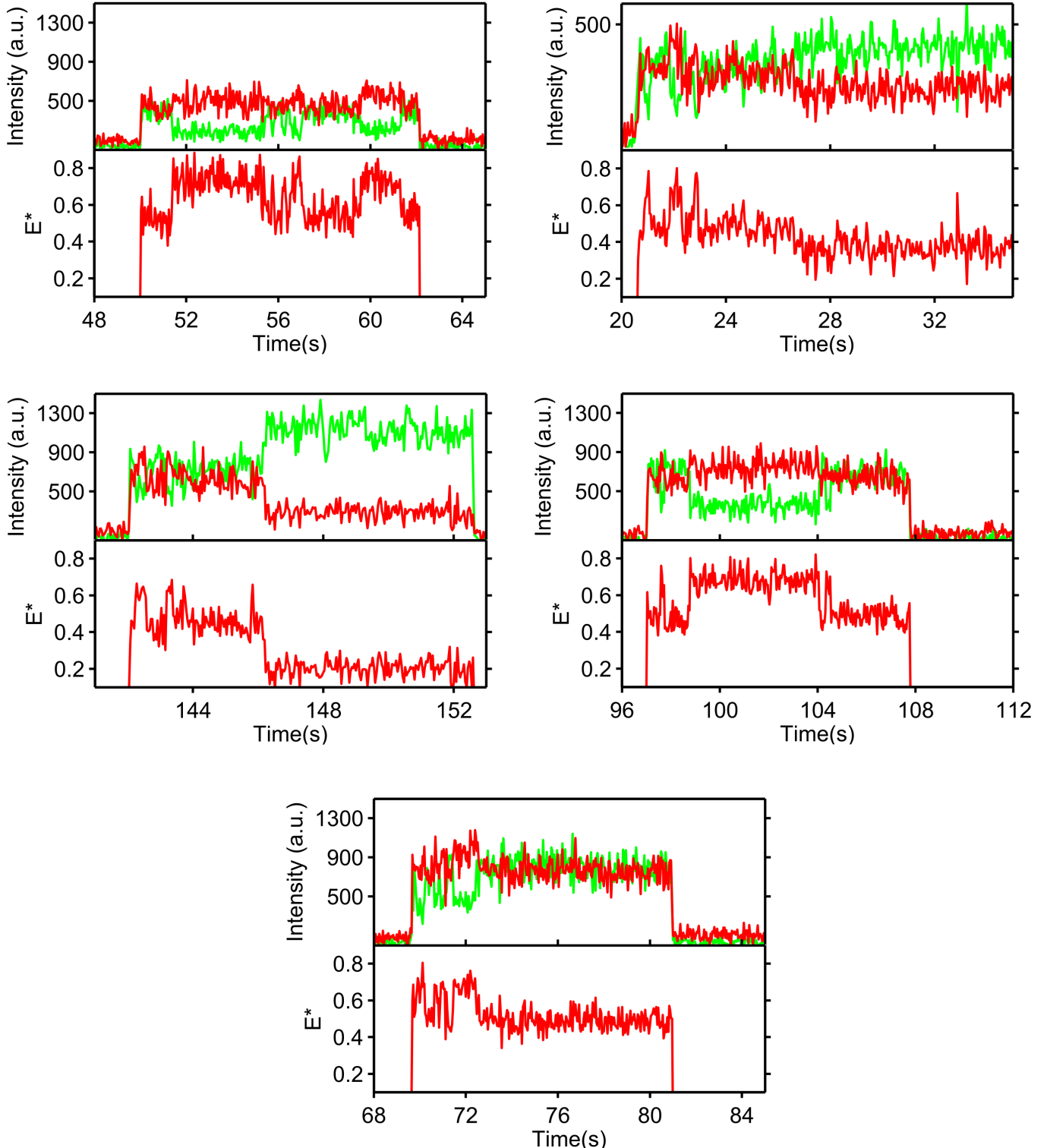
*

SI Figure 4. KF-DNA complexes in the presence of 0.25 µM dATP and dTTP to allow extension by 5-nt, with 250 µM of dCTP to suppress binary complex dynamics after polymerisation is complete, given it provides a mismatching nucleotide for template base 6. KF molecules are seen to interconvert between the open and closed conformations for a limited number of cycles, interpreted as incorporation of ~5 nt, with dwells in the fingers-open conformation caused by waiting for nucleotides to bind.

*
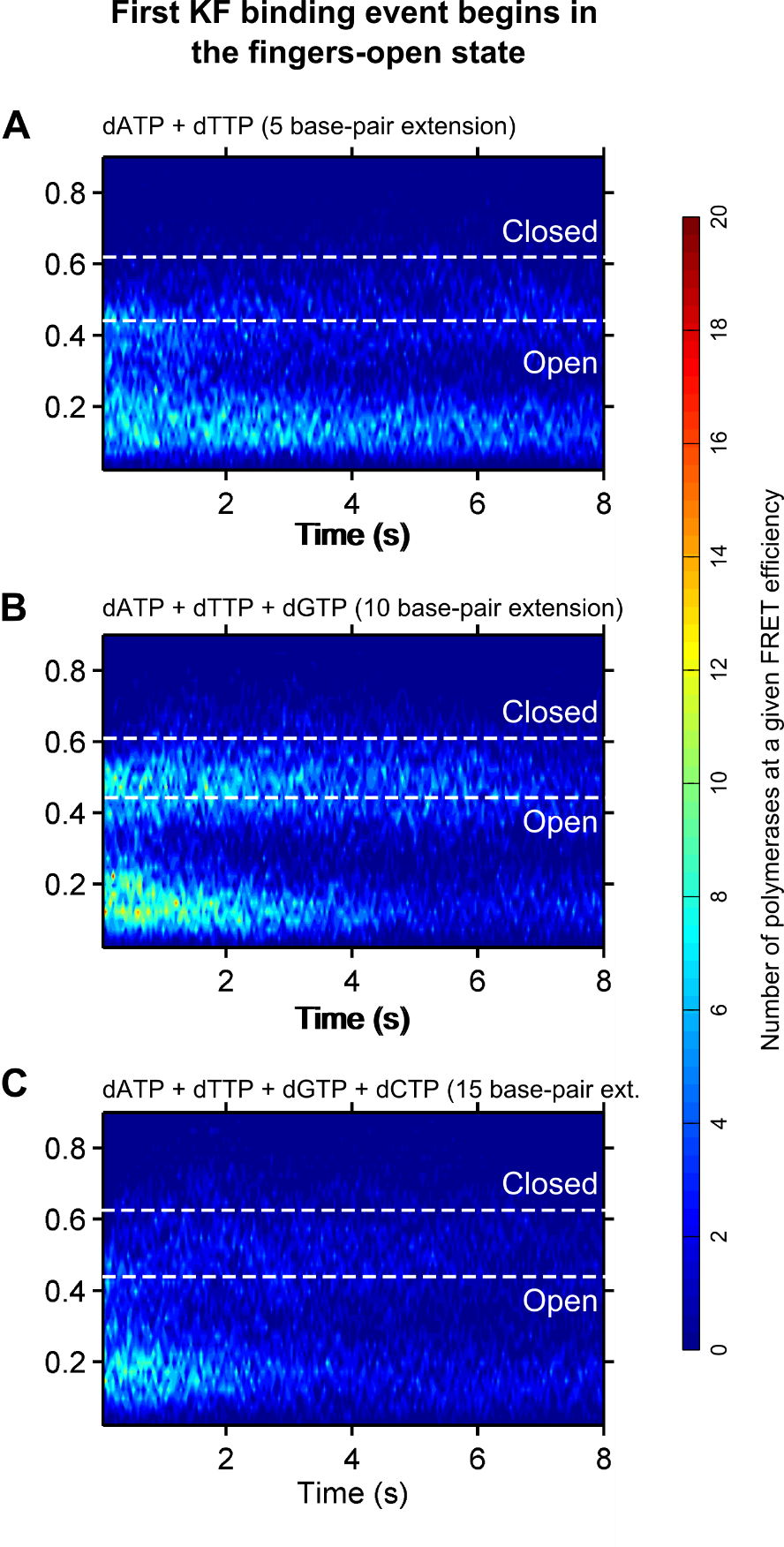
*

**SI Figure 5.** Colourmap of time-series histograms of the FRET efficiency of KF binding events, post synchronised across an entire sample. Remaining molecules from the experiment detailed in Figure 3, which begin in the fingers-open conformation. Conditions for DNA extension by maximally (A) 5 nucleotides (B) 10 nucleotides and (C) 15 nucleotides. The conformation of this population of KF molecules does not change significantly over time. Contours are used to aid visualisation; in reality, the data are quantised in 40-ms steps.

*
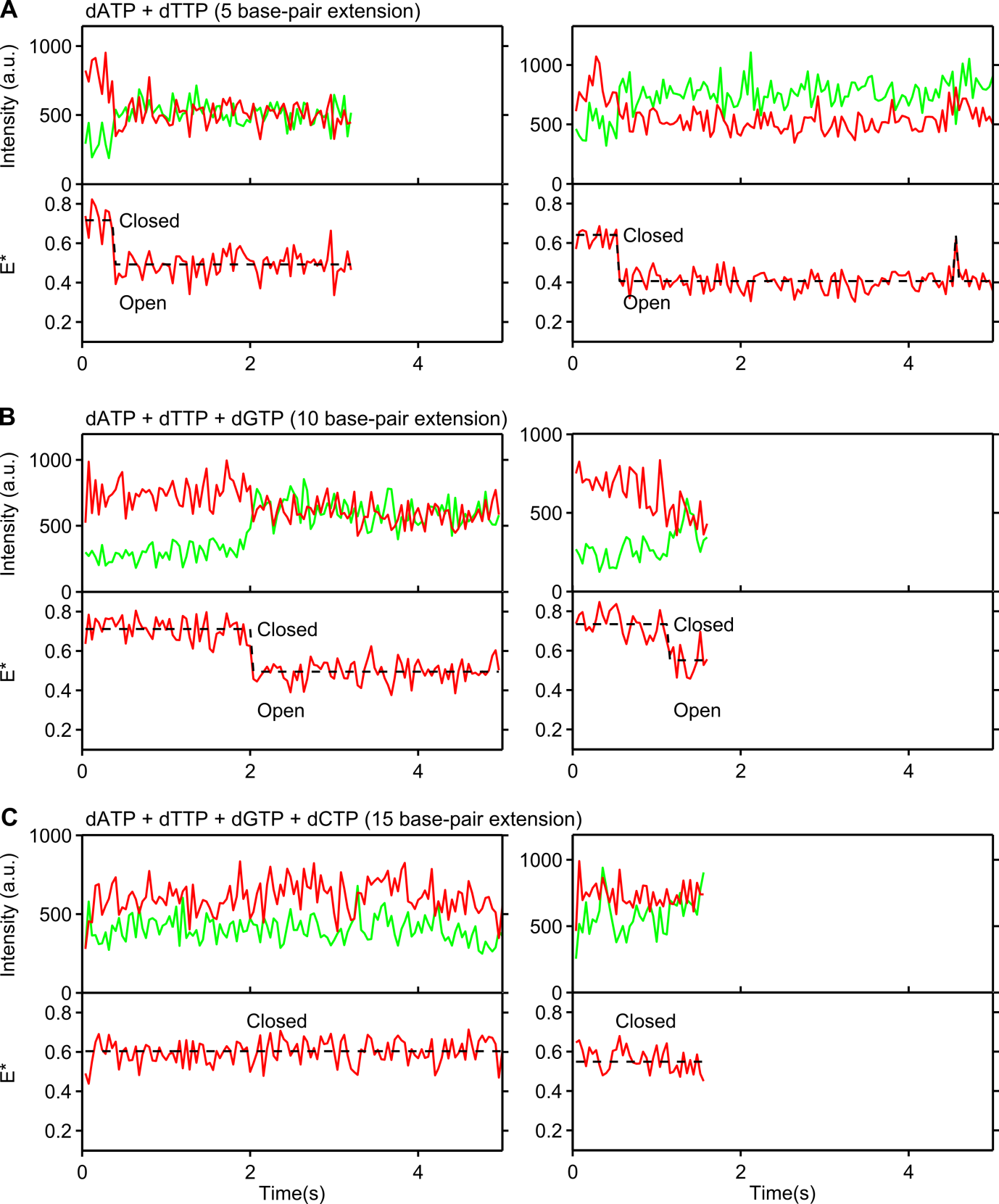
*

**SI Figure 6**. Sample HMM fits to KF-DNA binding events in the presence of A+T nucleotides (panel A), A+T+G nucleotides (panel B) and A+T+C+G nucleotides (panel C). The first dwell in the closed conformation corresponds to the polymerisation time.


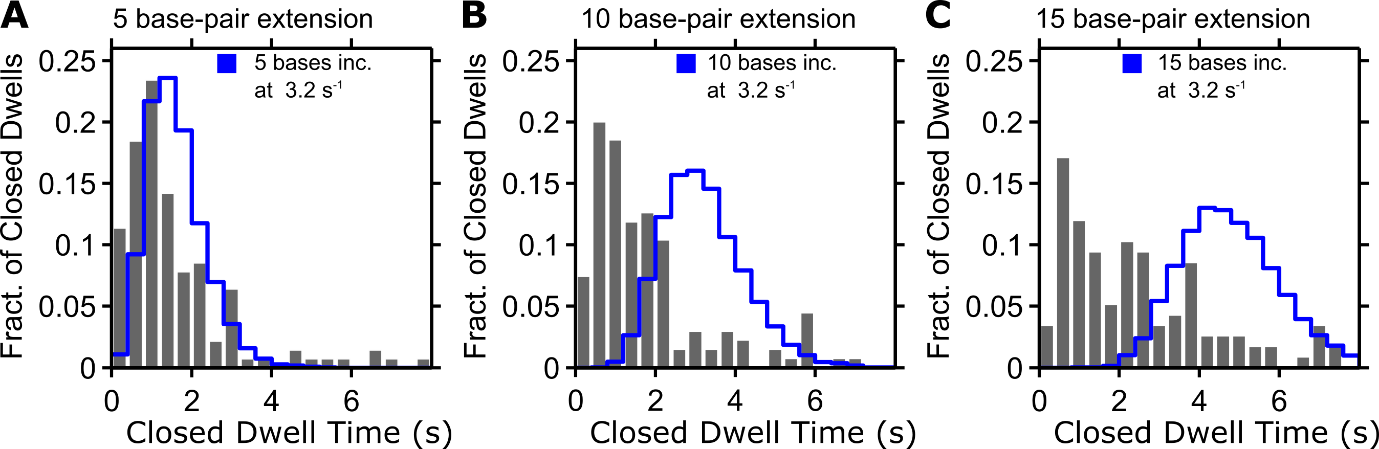


**SI Figure 7**. The first dwells in the fingers-closed conformation upon binding of KF to DNA in the presence of A+T, A+T+G, and A+T+G+C, allowing maximal extension by 5, 10 and 15 nucleotides, respectively (grey, panels A, B, and C), as per Fig. 4. Simulated models are superimposed in blue, in which all bases are assigned the same incorporation rate; 3.2 s^-1^. This shows reasonable fit to panel A, but not to panels B or C, demonstrating the measured polymerisation-times do not conform to a simple model in which all nucleotides are incorporated at the same rate.


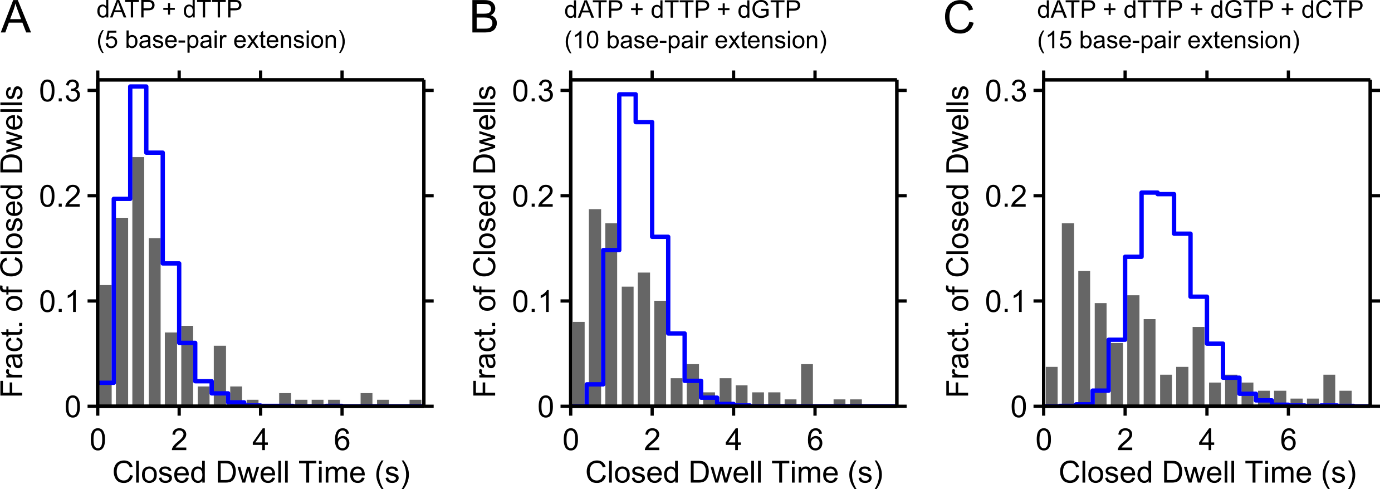


**SI Figure 8** As figure SI. 7, with nucleotides simulated to incorporate at equal rates, with the best fit chosen for each case. This extracts incorporation rates of 4, 5.8 and 5 s^-1^ to the distributions in panel A, B, and C respectively. While the incorporation rates are reasonably consistent, the form of the distributions for 10- and 15-base incorporations are significantly different from the measured distributions, suggesting that a model in which all nucleotides are incorporated equally is not an acceptable model.


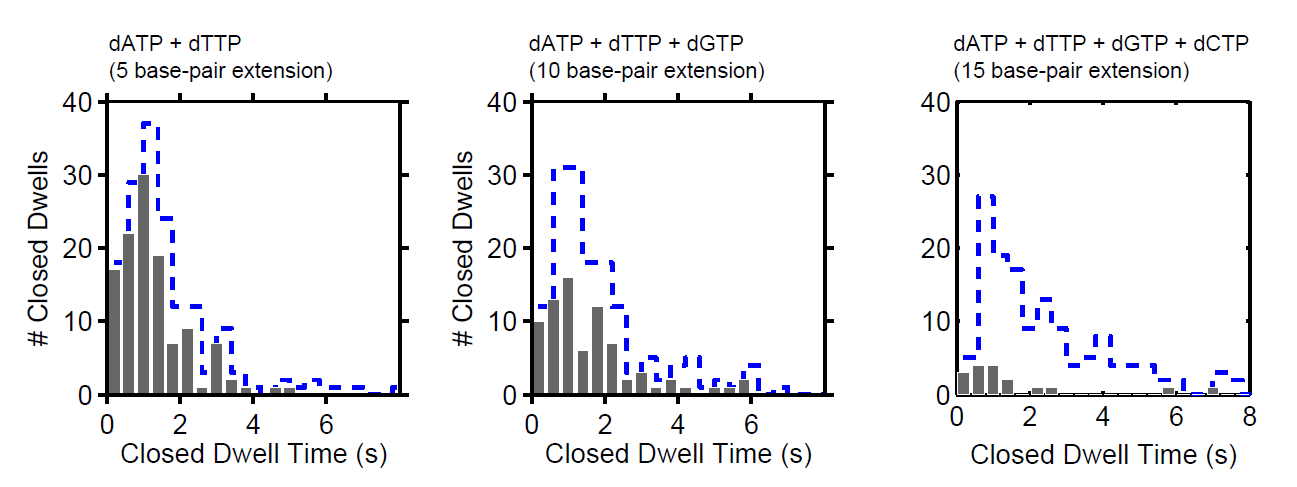


**SI Figure 9.** First dwells in the fingers-closed conformation upon binding of KF to DNA in the presence of A+T, A+T+G, and A+T+G+C, allowing extension by maximally 5, 10 and 15 nucleotides, respectively (panels A, B, C). Data and analysis as per Figure 4 for the blue dotted line. Grey bars represent length of dwells in the fingers-closed conformation, which subsequently are seen to return to the fingers-open conformation before dissociation of KF from the DNA.

*
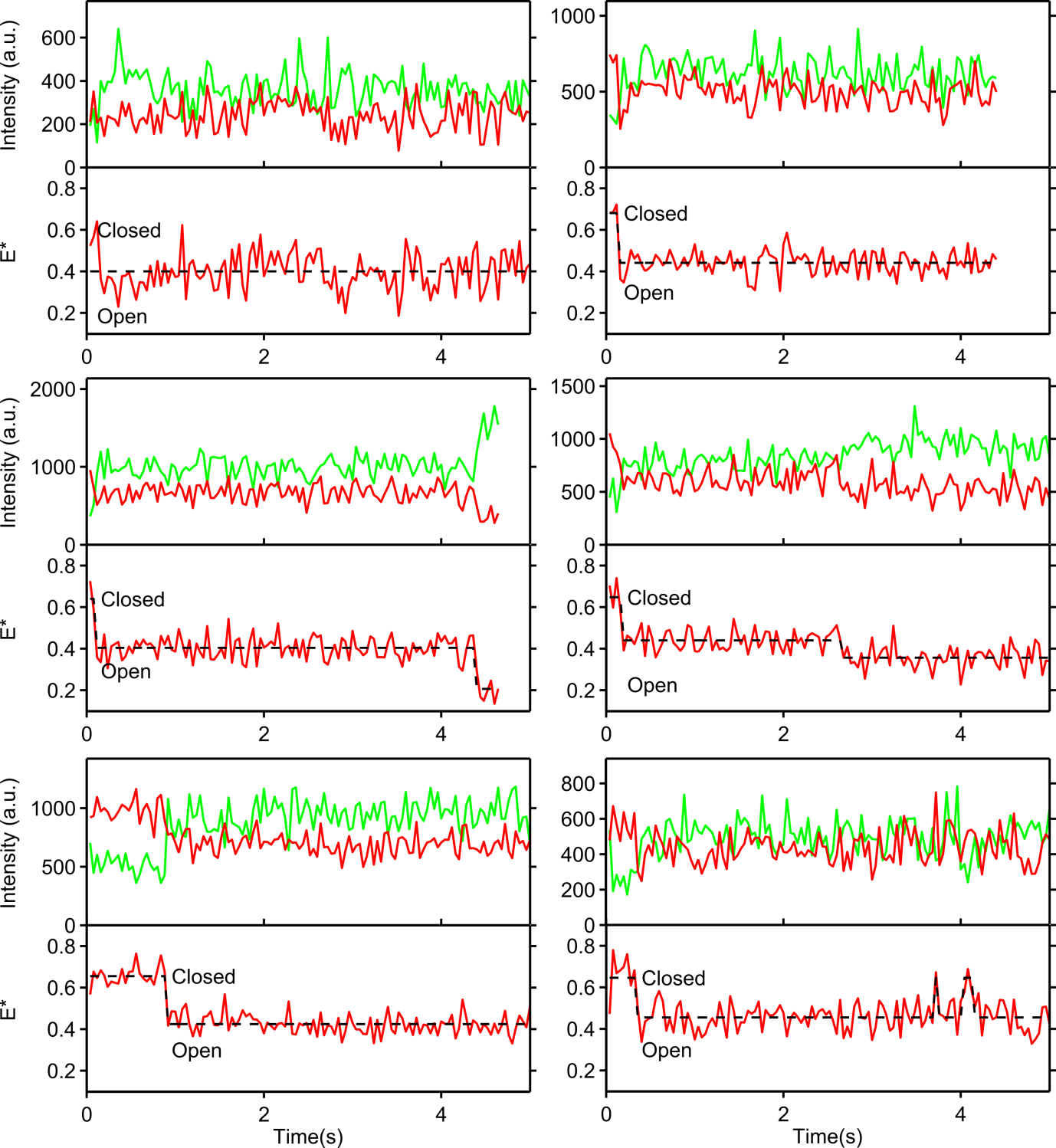
*

**SI Figure 10.** Sample HMM fits to KF-DNA binding events in the presence of dATP to induce single base incorporations. The first dwell in the closed conformation, corresponding with the polymerisation time. Note the upper left-hand panel has a closed state dwell not fit by the HMM, illustrating the importance of using two techniques to extract the incorporation rate.


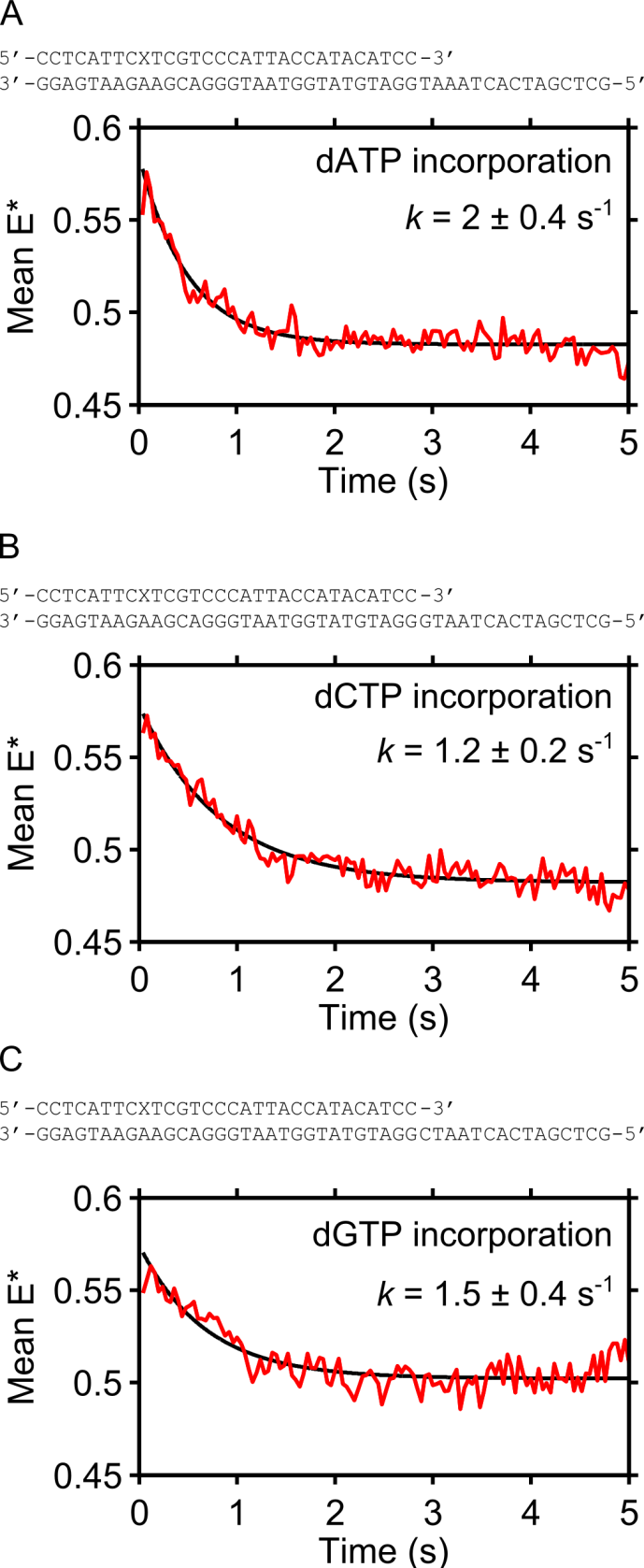


SI Figure 11. Mean E* of post-synchronised KF-DNA binding events across a collection of DNAs in the presence of complementary nucleotide (dATP, dCTP, and dGTP for panels A, B, and C, respectively). Mean E* fit to an offset single exponential to extract the single-nucleotide processive polymerisation rate for each nucleotide. Primer templates used in each experiment are denoted above each panel, with the first templating base as T, G and C for panels A, B, and C, respectively. X indicates labelling position with Cy5. Results are collated from KF binding events to 290, 322 and 346 DNA molecules for panels A, B and C, respectively.
